# Harvesting of olfactory ensheathing cells for autologous transplantation into the spinal cord injury. Its complexity in dogs

**DOI:** 10.3389/fnana.2015.00110

**Published:** 2015-08-25

**Authors:** Ignacio Salazar, William A. Barrios Santos, Alfonso Zubizarreta, Pablo Sánchez Quinteiro

**Affiliations:** ^1^Unit of Anatomy and Embryology, Department of Anatomy and Animal Production, Faculty of Veterinary, University of Santiago de CompostelaLugo, Spain; ^2^Unit of Otorhinolaryngology, Hospital Universitario Lucus AugustiLugo, Spain

**Keywords:** dog, nasal septum, lamina propria, olfactory glia, transplantation, spinal cord injuries

The mucosa lining the nasal cavity and its content contains olfactory sensory neurons (OSNs). The OSNs are organized into different territories (four in mammals with a more sophisticated model) and, with the exception of the OSNs enclosed in the vomeronasal organ, they are directly exposed to the external environment and are hence vulnerable to physical, chemical or traumatic injuries. This permanent exposure to toxic substances is probably the reason why the olfactory epithelium has developed the ability to continually replace neurons (Graziadei and Graziadei, 1979).

Due to neurogenesis, which persists even through adulthood, there are neurons in the OE with different degrees of development. The apical end of the mature neurons detects odorants trough the cilia of the corresponding dendrites, and from the basal side of such neurons an axon projects outside the OE into the lamina propria (LP). Axons from different adult neurons form bundles and later nerves, olfactory nerves, which cross the cribriform plate of the ethmoid bone and meninges reaching the olfactory nerve and glomerular layers of the olfactory bulb. From the LP to their target, only the olfactory ensheathing cells (OECs) enwrap these unmyelinated olfactory axons preventing them from having contact with any other kind of glial cells (Ramón-Cueto and Avila, 1998). OECs have emerged as one of the most promising candidates to repair nervous system lesions, specifically spinal cord injuries (SCIs) (Raisman et al., 2012), although this is a matter of not little controversy, due to methodological concerns and deficits in experimental designs (Granger et al., 2014).

Methodologically, autologous transplantation implies serious difficulties, and the adequate taking of tissue samples is an important prerequisite to define. To date, different species have been used to evaluate the transplantation of OECs and the dog is thought to be an interesting model, among other reasons, because (i) SCI occurs naturally (Bergknut et al., 2013), (ii) some true trial has been successfully done (Granger et al., 2012), (iii) the size and peculiarities of their olfactory tissue (Ziege et al., 2013), and (iv) the morphological proximity of their vertebral column and spinal cord to humans'. In dogs the LP is especially well developed in the olfactory mucosa (OM) of the posterior part of the nasal septum, near the cribriform plate of the ethmoid bone, and that is therefore the most favorable area to harvest OECs (Figure 1). Because there are different structures constituting the OM (Table 1, in Supplementary Material) and because of the unknown role these structures could play in transplantation, it has been suggested that when taking samples, the whole OM must be included (Lindsay et al., 2010).

**Figure 1:**
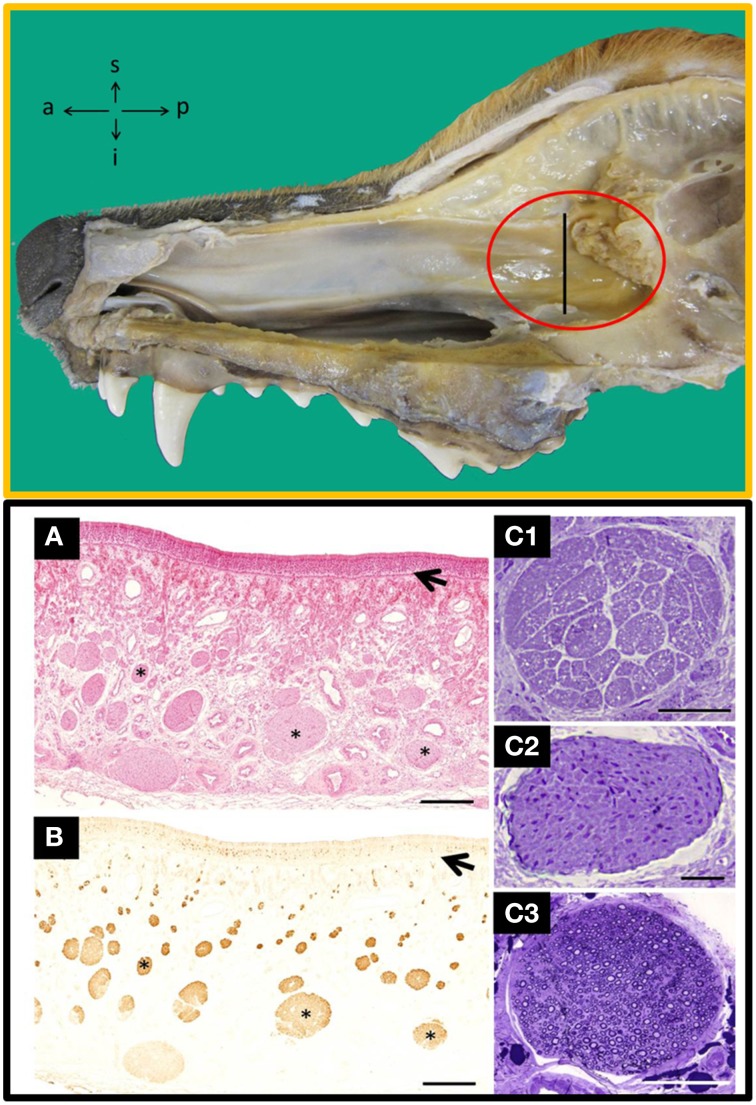
**Top:** Inside view of the nasal cavity showing the nasal septum where the area of olfactory mucosa has the highest concentration of nerves bundles (red circle). a, anterior; i, inferior; p, posterior; s, superior. **Bottom**: Transverse sections of the nasal septum, at the level indicated by the perpendicular line in the TOP figure stained with haematoxylin-eosin **(A)** and anti-OMP **(B)**, a specific marker for olfactory tissue; arrows indicate the basal lamina and asterisks nerve bundles. Semithin transverse sections of nerves bundles likely to be found in the lamina propria from: unmyelinated olfactory nerves **(C**_**1**_**)**, unmyelinated vomeronasal nerves **(C**_**2**_**)** and branches of the myelinated trigeminal nerves **(C**_**3**_**)**. Scale bars: **(A,B)**, 500 μm; **(C**_**1**_**–C**_**3**_**)**, 50 μm. [Top and Bottom **(A,B)**, figures previously published in their original form or slightly adapted from Barrios et al. (2014). Bottom **(C)**, comes from a doctoral dissertation published by the Spanish Ministry of Education (1995)].

Reaching the suggested target is quite complicated in dogs because of the complexity of their nasal conchae. Nasal conchae of dogs fill the nasal cavity almost entirely in a considerable extension of its total length, and as a consequence the common nasal meatus is truly a very narrow space, clearly insufficient to practice the necessary nasal endoscope-assisted microsurgical dissection. An alternative would be to go for the transnasal/transseptal/submucoperiosteal microsurgical approach, which is a part of the traditional transnasal/transsphenoidal approach for treatment of pituitary tumors in humans. This technique involves a total or partial destruction of the ventral and middle nasal conchae and the ethmoturbinates in dogs (Figures S1–S3, in Supplementary Material), and the side effects must be rigorously assessed.

The nasal septum is not the only component of the olfactory system to be lined by OM. The region where OM covers the endoturbinates II and III is a zone where the epithelium and the LP are importantly developed, and is therefore a liable area to be considered as an alternative source of OECs (Barrios et al., 2014). Nevertheless, the endoturbinates are delicate bony scrolls and it is not recommended to isolate mucosa from them. Added to that, the access to the endoturbinates will encounter the same difficulties than the access to the nasal septum.

The ectoturbinates are structured in a slightly different way than the endo, as they have a smaller degree of density/concentration, are more fragile and their OM is less developed, but the direct access to them may also be of great difficulty. However, an interesting characteristic of this group of ethmoturbinates concerns its topography. The ecto 2 and 3 project to the rostral and medial parts, and lateral part of the frontal sinus (FS), respectively, (Figure S4, in Supplementary Material). The FS is theoretically the most accessible path to the OM. Nonetheless, regardless of whether the OM associated to the ectoturbinates is the most appropriate to get samples of OECs, there is a critical point that must be taken into account. The FS of dogs, usually integrated by three parts (lateral, rostral, and medial), shows an enormous variability, to the extent that there are not two identical frontal sinuses, and even within the same subject symmetry is not found between the left and right side. This latter feature suggests that breed is not an overwhelmingly determinative issue to justify such variability. In the dissection room we have observed significant changes in the general conformation of the FS, although in our previous studies (Barrios et al., 2014) we have focused our attention in adult mesaticephalic dogs, that is to say German Shephersds or mongrels derived therefrom. The most significant changes concern the FS and its compartments, and hence its form and topography, and the amount or size of the ectoturbinates projected to the sinus which is highly variable, or even absent in some cases. Moreover, the mucosa lining the sinus itself does not have the typical conformation of the OM. All of these facts advise against the consideration of the frontal sinus—its mucosa and/or its contents—as the ideal place to harvest OECs in adult dogs.

## Concluding remarks

The potential efficacy of OECs to repair SCIs through autologous transplantation requires a feasible approach. When harvesting these cells it is highly recommended to collect full parts of the OM, and even proceeding this way, the extraction of OM for autologous transplantation in dogs bears some difficulties. In order to avoid confusion and misinterpretation, any technique used to harvest OECs in dogs must be carefully evaluated and described to ensure its susceptibility to be repeated as many times as necessary, which is a basic principle in research. The possibility of taking samples of OECs directly from the olfactory bulb as it is done in human medicine (Czyz et al., 2015) should be also objectively explored in dogs.

### Conflict of interest statement

The authors declare that the research was conducted in the absence of any commercial or financial relationships that could be construed as a potential conflict of interest.

## Additional information

In our lab, the work that we do is in accordance with the laws and regulations of the European Union (2010/63UE) and Spain (RD 53/2013) for the care and handling of animals in research, and with the guidelines of the University of Santiago de Compostela Bioethical Committee.
