# Supplementary material for: Harvesting of olfactory ensheathing cells for autologous transplantation into the spinal cord injury. Its complexity in dogs
Source: Front Neuroanat. 2015 Aug 25;9:110. doi: 10.3389/fnana.2015.00110 (PMC4548204; doi:10.3389/fnana.2015.00110)
Supplement: Supplementary file 1 [file Presentation1.PDF]

Frontiers in Neuroanatomy

Supplementary Material

Harvesting of olfactory ensheathing cells for autologous transplantation into the spinal cord injury. Its complexity in dogs

Ignacio Salazar[1]*, William A. Barrios Santos[1†], Alfonso Zubizarreta[2], Pablo Sánchez[1]

[1] *Department of Anatomy and Animal Production, Unit of Anatomy and Embryology, Faculty of Veterinary, University of Santiago de Compostela, Lugo, Spain*

[2] *Hospital Universitario Lucus Augusti, Unit of Otorhinolaryngology, San Cibrao s/n, 27003 Lugo, Spain*

*Correspondence:

Ignacio Salazar, Department of Anatomy and Animal Production, Unit of Anatomy and Embryology, Faculty of Veterinary, University of Santiago de Compostela, Av Carballo Calero s/n, 27002 Lugo, Spain.

e-mail: ignacio.salazar@usc.es

[†] Present Address:
William A. Barrios Santos,
Department of Physiology,
Universidad Nacional de San Cristóbal de Huamanga, Ayacucho, Perú

Table 1

- **OLFACTORY MUCOSA**
  - **EPITHELIUM**
    - Sustentacular cells
    - Neurons ....
      - mature
      - immature
    - Basal cells ....
      - globose
      - horizontal
    - Bowmann's gland .... excretory canals
  - -----------------basal lamina-----------------
  - **LAMINA PROPRIA**
    - Connective tissue ..... different cells
    - Bowman's gland ...... excretory canals
    - Olfactory ensheathing cells and mesenchymal stem cells
    - Collagen & extracellular matrix
    - Vessels ....
      - smooth muscle cells
      - endothelial cells
    - Nerve bundles ....
      - olfactory
      - vomeronasal
      - trigeminal

Table 1. Summary of the components that make up the olfactory mucosa. A basal lamina establishes the limit between the epithelium and the lamina propria.

Figure S1

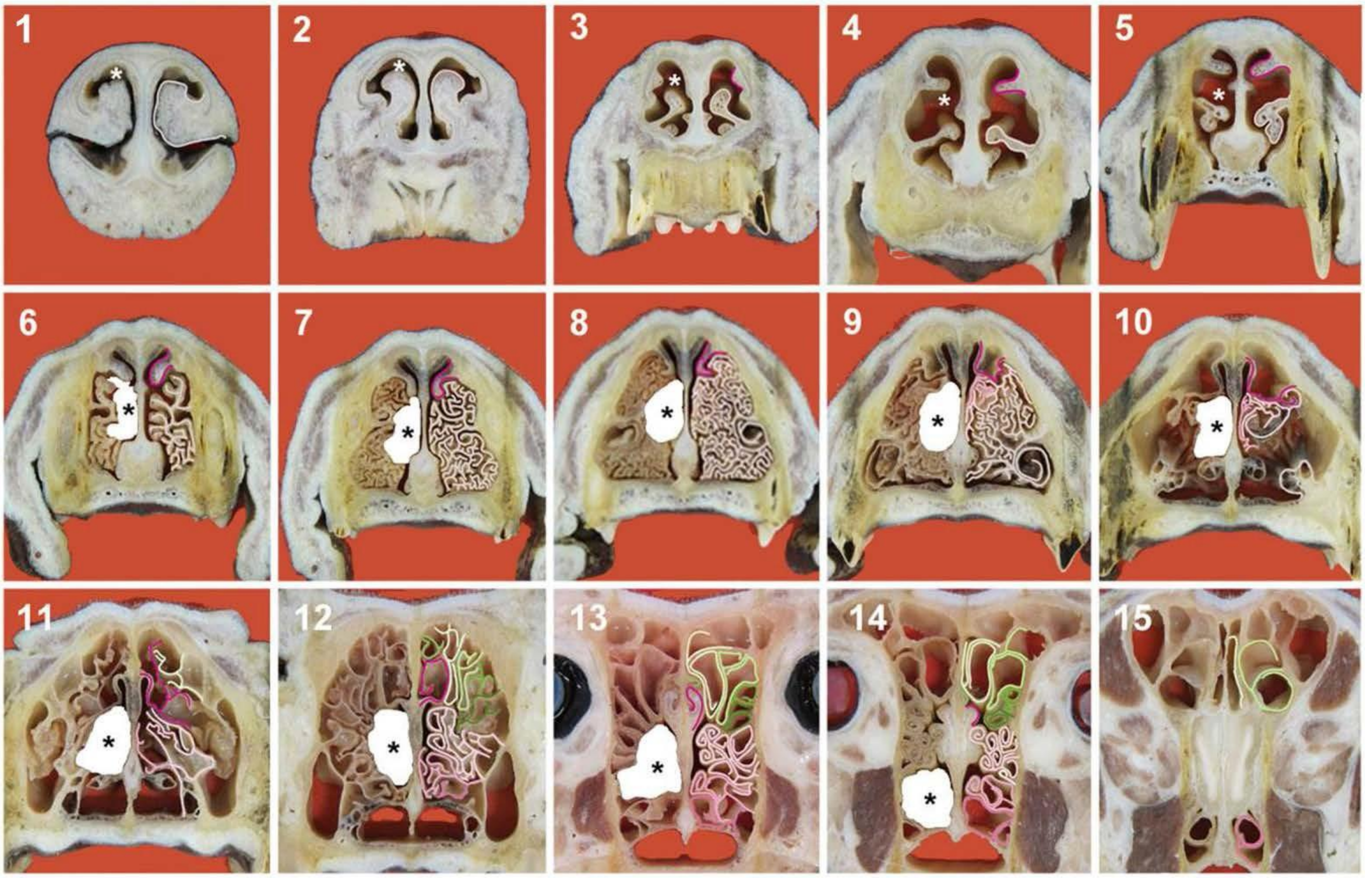

Fig. S1. Transverse frozen sections of the nasal cavity of an adult mesaticephalic dog from anterior (1) to posterior (15). On the left side of each photo the asterisks indicate the way to follow when reaching the selected olfactory mucosa. On the right side, the nasal conchae and the ethmoturbinates are identified as specified in figure S2. (Previously published in their original form or slightly adapted from Barrios et al., 2014 Front Neuroanat2014, 8:106.doi: 10.3389/fnana.2014.0010).

Figure S2

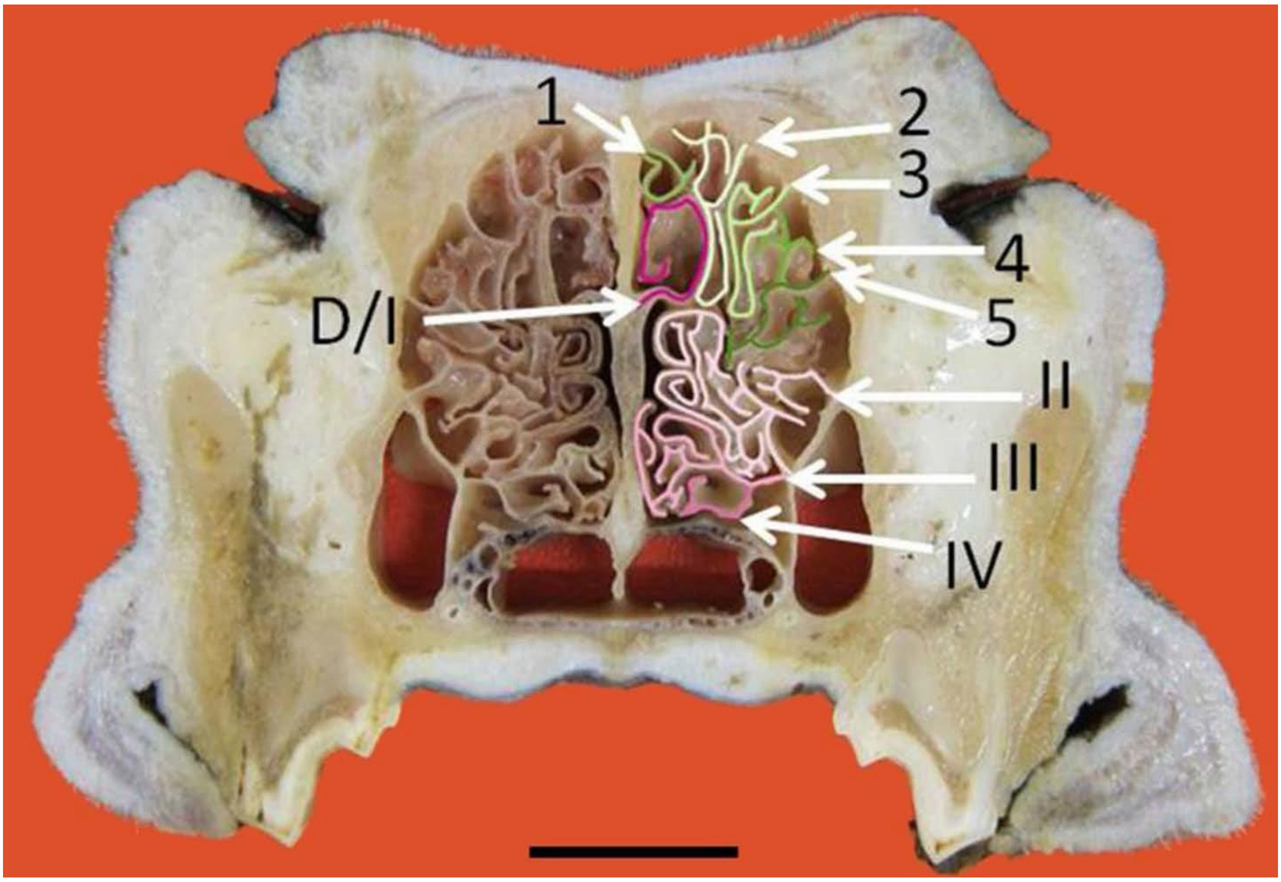

Fig. S2. Transverse frozen sections of the posterior third of the nasal cavity used as a guidance model for the identification of nasal conchae and ethmoturbinates.D, dorsal nasal concha.Ectoturbinates are identified by Arabic numerals (1-5) and endoturbinates by roman numerals (II-IV). Scale bar, 2cm. (Previously published in their original form or slightly adapted from Barrios et al., 2014 Front Neuroanat2014, 8:106.doi: 10.3389/fnana.2014.0010).

Figure S3

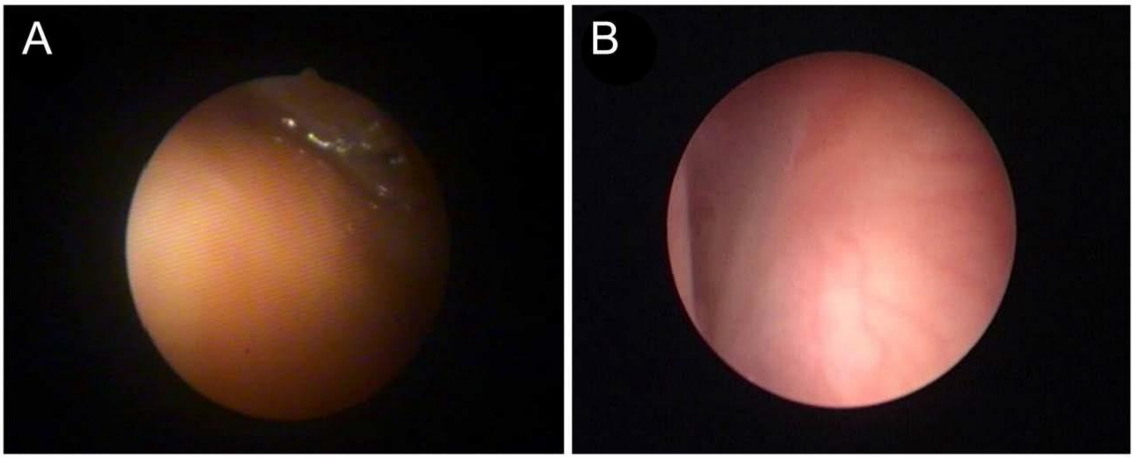

Fig. S3. The total or partial destruction of conchae/ethmoturbinates allows visualizing by endoscopy the appropriate area of the olfactory mucosa (A), clearly differentiated from the respiratory mucosa (B), and also allows practicing the corresponding tissue biopsy. (Data source from Cifuentes et al., 2011. Anatomía Veterinaria. Unicopia Artes Gráficas S.L. Lugo).

Figure S4

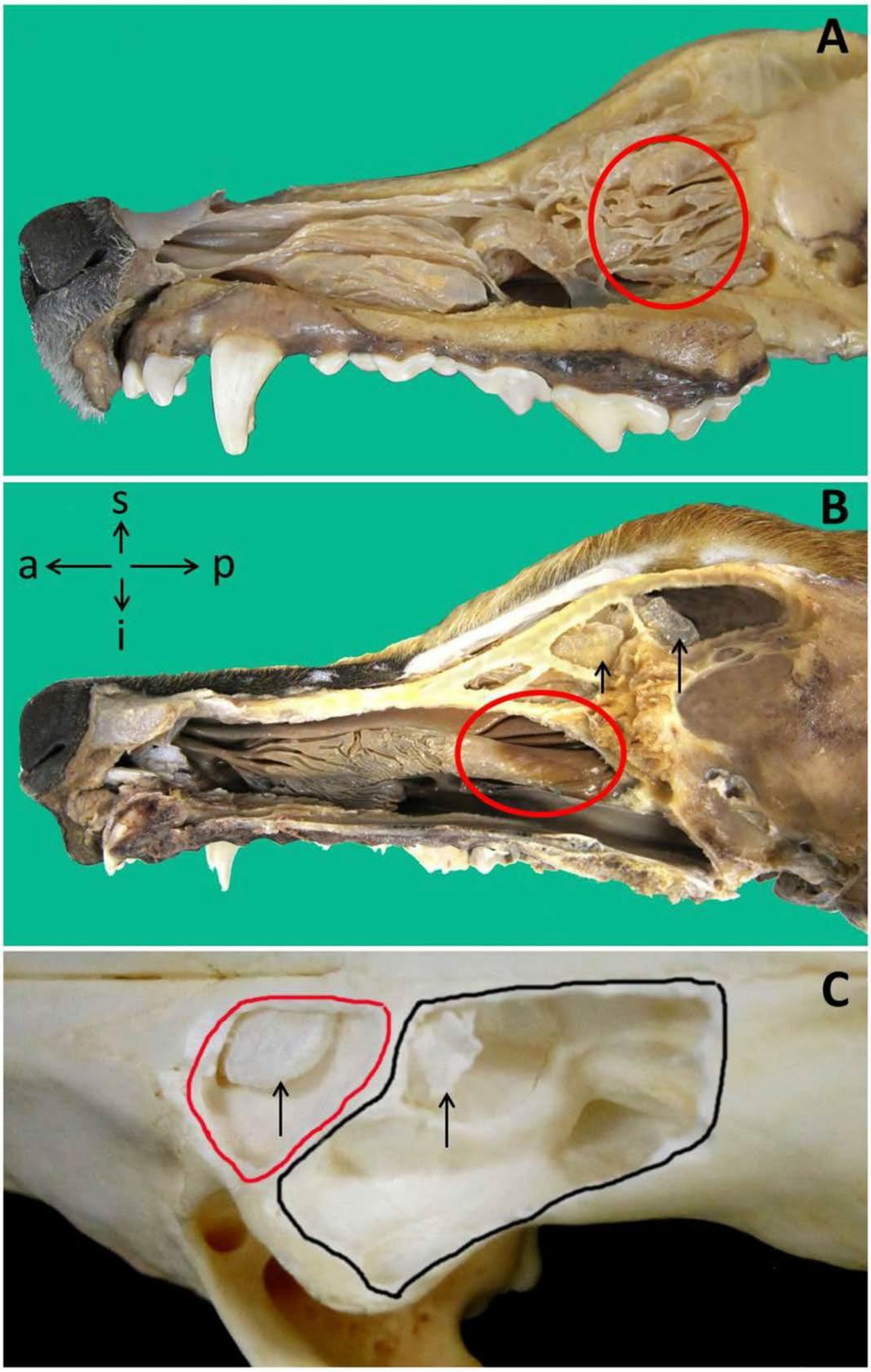

Fig. S4. Lateral (A) and medial (B) views of the turbinate complex in the adult dog. The olfactory mucosa is located in the posterior part of the ethmoturbinates (red circles), some of them projecting to the frontal sinus (arrows in B). Superior view of the frontal sinus in the head skeleton (C) showing the projection of the ectoturbinates 2 and 3 on it (arrows).a, anterior; i, inferior; p, posterior; s, superior. (A & B previously published in their original form or slightly adapted from Barrios et al., 2014 Front Neuroanat2014, 8:106. doi: 10.3389/fnana.2014.0010; C data source from Cifuentes et al., 2011. Anatomía Veterinaria. Unicopia Artes Gráficas S. L. Lugo).
